# Mitochondrial Adaptations to Exercise Training in Equine Skeletal Muscle: A Narrative Review

**DOI:** 10.3390/life16061008

**Published:** 2026-06-16

**Authors:** Vlad Cocioba, Paula Nistor, Daniel George Bratu, Șerban Blaga, Bianca Cornelia Zanfira, Călin Mircu, Ioan Huțu

**Affiliations:** 1 Horia Cernescu Research Unit, Faculty of Veterinary Medicine Timisoara, University of Life Science “Regele Mihai I” from Timisoara, Calea Aradului 119, 300645 Timisoara, Romania; vlad-mihai.cocioba.fmv@usvt.ro (V.C.); daniel.bratu@usvt.ro (D.G.B.); serban.blaga.fmv@usvt.ro (Ș.B.); bianca.lungu@usvt.ro (B.C.Z.); calinmircu@usvt.ro (C.M.); ioan.hutu@fmvt.ro (I.H.); 2 Doctoral School “Veterinary Medicine”, University of Life Sciences “King Mihai I” from Timişoara, Calea Aradului 119, 300645 Timişoara, Romania; 3 Department of Infectious Diseases and Preventive Medicine, Faculty of Veterinary Medicine, University of Life Sciences “King Mihai I” from Timişoara, 300645 Timişoara, Romania; 4 Extension Unit and Advisory Center, 300645 Timisoara, Romania

**Keywords:** equine exercise physiology, mitochondria, skeletal muscle, oxidative phosphorylation, mitochondrial biogenesis, metabolic flexibility, equine athletes, exercise adaptation

## Abstract

The horse represents one of the most physiologically specialized athletic mammals, capable of sustaining both high-intensity and prolonged exercise. Central to this remarkable performance capacity is the metabolic adaptability of skeletal muscle and its mitochondrial network. This narrative review synthesizes current evidence from equine, human, and rodent studies on exercise-induced mitochondrial remodeling in equine skeletal muscle. A comprehensive literature search was conducted across PubMed, Web of Science, and Scopus using terms related to equine exercise physiology, mitochondrial biology, and skeletal muscle metabolism. Preference was given to peer-reviewed original research and review articles. Mitochondria regulate oxidative phosphorylation, substrate oxidation, redox signaling, and cellular responses to metabolic stress induced by exercise. Training induces extensive mitochondrial adaptations, including mitochondrial biogenesis, remodeling of the respiratory chain, enhanced oxidative phosphorylation efficiency, and increased metabolic flexibility. These adaptations are believed to contribute to improvements in aerobic capacity, delayed fatigue onset, and enhanced recovery following exercise, although direct mechanistic evidence in horses remains limited. In equine skeletal muscle, mitochondrial plasticity is closely linked to muscle fiber composition and the distribution of oxidative and glycolytic fibers. Exercise-induced signaling pathways involving AMP-activated protein kinase (AMPK), Ca^2+^-dependent kinases, and the transcriptional coactivator PGC-1α regulate mitochondrial biogenesis and metabolic remodeling. In addition, mitochondrial dynamics, including fusion, fission, and mitophagy, maintain mitochondrial quality and functional efficiency during repeated training stimuli. Experimental studies in Thoroughbred and Standardbred horses demonstrate that training has been associated with increases in mitochondrial density and respiratory capacity in equine skeletal muscle, contributing directly to improved aerobic performance and metabolic efficiency. However, mitochondrial adaptations must be interpreted within the broader context of musculoskeletal adaptation, as metabolic improvements may occur faster than structural adaptation of tendons and ligaments. This review synthesizes current knowledge on exercise-induced mitochondrial remodeling in equine skeletal muscle, while highlighting the limited mechanistic evidence available in horses and the need for more standardized longitudinal studies.

## 1. Introduction

The horse (*Equus caballus*) represents one of the most remarkable examples of physiological specialization for athletic performance among mammals. Through centuries of selective breeding aimed at maximizing speed, endurance, and power output, modern sport horses exhibit important cardiovascular, respiratory, and musculoskeletal capacities that enable them to sustain intense physical activity [1,2,3]. During maximal exercise, oxygen consumption in horses can increase dramatically compared with resting conditions, reflecting a good ability to deliver and utilize oxygen at the level of working muscles [4,5].

Skeletal muscle is therefore the primary site of energy expenditure during exercise, and its metabolic characteristics largely determine the athletic capacity of the animal [5]. Muscle contraction requires a continuous supply of adenosine triphosphate (ATP) to support cross-bridge cycling, the maintenance of ionic gradients, and calcium handling within muscle cells [6]. The rapid turnover of ATP during intense exercise demands efficient metabolic systems capable of sustaining energy production under fluctuating workloads [1].

Mitochondria play a central role in this process. As the main site of oxidative phosphorylation, mitochondria generate the majority of ATP during aerobic exercise. In addition to energy production, mitochondria participate in multiple regulatory functions including substrate metabolism, redox signaling, apoptosis, and intracellular calcium homeostasis [7,8]. These functions place mitochondria at the intersection of metabolic regulation and cellular adaptation to physiological stress.

In skeletal muscle, mitochondrial characteristics collectively define what has been termed the mitochondrial phenotype. This concept encompasses mitochondrial density, respiratory capacity, substrate utilization efficiency, and the mechanisms responsible for mitochondrial quality control. Importantly, the mitochondrial phenotype is highly plastic and can be modified in response to environmental stimuli, particularly exercise training [9,10].

Exercise training induces extensive mitochondrial remodeling in skeletal muscle. These adaptations include increased mitochondrial biogenesis, improved efficiency of oxidative phosphorylation, enhanced fatty acid oxidation, and the reinforcement of antioxidant defense mechanisms. Together, these changes allow skeletal muscle to sustain prolonged exercise, delay the onset of fatigue, and recover more rapidly following intense physical activity [11,12].

In horses, mitochondrial plasticity is particularly relevant because locomotor muscles operate near their physiological limits during intense exercise. Racing and endurance competitions impose significant metabolic stress, requiring efficient coordination of energy production pathways to maintain performance [9,13].

Although significant progress has been made in understanding mitochondrial biology in human and rodent models, research focusing specifically on mitochondrial adaptations in equine skeletal muscle remains comparatively limited. Nevertheless, available studies suggest that mitochondrial remodeling plays a crucial role in determining the athletic capacity of horses [5,9,14].

In recent years, increasing attention has been directed toward understanding the molecular basis of exercise-induced mitochondrial remodeling in skeletal muscle. Identifying the signaling pathways and metabolic adaptations that regulate mitochondrial function may provide valuable insights for optimizing training programs and improving athletic performance in sport horses [9,12].

Despite growing interest in equine exercise physiology, our understanding of mitochondrial adaptations to exercise in equine skeletal muscle remains comparatively limited and fragmented. A major limitation in the field is the methodological heterogeneity across studies. Variability in training protocols, exercise intensity, duration, muscle sampling sites, and analytical techniques complicates direct comparisons and limits the ability to draw generalized conclusions [10,14]. Another important gap lies in the limited mechanistic insight into the signaling pathways governing mitochondrial adaptations in equine muscle. While key regulators such as AMP-activated protein kinase (AMPK), calcium/calmodulin-dependent protein kinase (CaMK), p38 mitogen-activated protein kinase (p38 MAPK), and peroxisome proliferator-activated receptor gamma coactivator-1 alpha (PGC-1α) are well characterized in other species, direct evidence of their coordinated regulation and functional significance in horses remains sparse [9,10]. The concept of the mitochondrial phenotype provides a valuable framework for understanding muscle plasticity; however, it has not been comprehensively characterized in horses [7]. In addition, the role of muscle fiber type composition in shaping mitochondrial responses to exercise warrants further investigation in the equine model. From an applied perspective, a deeper understanding of mitochondrial adaptations has important implications for training optimization, performance enhancement, and injury prevention in sport horses [13,14].

This review aims to synthesize current knowledge regarding mitochondrial adaptations to exercise in equine skeletal muscle. Particular attention is given to molecular signaling pathways regulating mitochondrial biogenesis, the structural and functional characteristics of equine muscle relevant to mitochondrial metabolism, metabolic flexibility during exercise, and the implications of these adaptations for athletic performance and training management. Future research should prioritize species-specific investigations, methodological standardization, and mechanistic depth to fully elucidate the role of mitochondria in equine exercise adaptation. Such efforts will be essential for advancing both fundamental knowledge and practical applications in equine sports science.

Throughout this review, a distinction is made between acute exercise responses and chronic training adaptations. Acute exercise responses refer to the immediate metabolic, redox, and signaling events triggered by a single exercise bout, whereas training adaptations reflect cumulative structural and functional changes resulting from repeated exercise exposure over time. Because the majority of available equine studies investigate adaptations occurring after weeks or months of conditioning, the present review primarily focuses on training-induced mitochondrial remodeling.

It is important to recognize that acute exercise and exercise training do not produce identical mitochondrial responses. A single bout of exercise primarily elicits transient metabolic perturbations, reactive oxygen species production, substrate depletion, and activation of intracellular signaling pathways. In contrast, repeated exercise exposure over time induces cumulative adaptations including mitochondrial biogenesis, remodeling of oxidative capacity, enhanced substrate utilization, and long-term improvements in mitochondrial function. Throughout this review, references to mitochondrial adaptation primarily concern chronic training-induced responses unless acute exercise effects are specifically discussed [7,10,13,14].

## 2. Literature Search Strategy and Scope of the Review

This study was conducted as a narrative review aimed at synthesizing current knowledge on exercise-induced mitochondrial adaptations in equine skeletal muscle.

### 2.1. Databases and Search Period

A comprehensive literature search was performed in the following electronic databases: PubMed/MEDLINE, Web of Science Core Collection, Scopus, and CAB Abstracts. Google Scholar was used as a supplementary resource to identify additional relevant publications not captured by the primary databases. The search was conducted between 2 December 2025 and 5 April 2026 and was not restricted by publication date in order to ensure the inclusion of foundational studies that have shaped the field.

### 2.2. Search Terms

The following search terms were used individually and in combination using Boolean operators (AND, OR):

Equine-specific terms: “horse” OR “equine” OR “Equus caballus” OR “Thoroughbred” OR “Standardbred” OR “Arabian”.

Skeletal muscle terms: “skeletal muscle” OR “locomotor muscle” OR “gluteus medius” OR “muscle fiber”.

Mitochondrial biology terms: “mitochondria” OR “mitochondrial biogenesis” OR “mitochondrial function” OR “oxidative phosphorylation” OR “electron transport chain” OR “mitochondrial dynamics” OR “mitophagy” OR “mitochondrial phenotype” OR “citrate synthase” OR “high-resolution respirometry”.

Exercise physiology terms: “exercise” OR “training” OR “exercise adaptation” OR “athletic performance” OR “endurance” OR “racing” OR “fatigue”.

Molecular signaling terms: “PGC-1α” OR “AMPK” OR “CaMK” OR “NRF” OR “TFAM” OR “metabolic flexibility” OR “reactive oxygen species”.

Additional targeted searches were conducted using specific combinations of equine and mechanistic terms to capture studies addressing particular aspects of the topic (e.g., “equine” AND “high-resolution respirometry”; “horse” AND “citrate synthase” AND “training”).

### 2.3. Inclusion and Exclusion Criteria

The following inclusion criteria were applied: (1) original research articles, review articles, and meta-analyses published in peer-reviewed journals; (2) studies investigating mitochondrial structure, function, biogenesis, dynamics, or quality control in skeletal muscle in the context of exercise; (3) studies examining molecular signaling pathways relevant to exercise-induced mitochondrial adaptation; (4) studies on metabolic substrate utilization during exercise in skeletal muscle; and (5) publications in English. Studies conducted in horses were prioritized. Studies in humans or rodent models were included when they provided mechanistic insights not yet available in equine research, and are explicitly identified as non-equine evidence throughout the text.

The following exclusion criteria were applied: (1) studies exclusively addressing non-skeletal muscle tissues without comparative relevance; (2) conference abstracts without full-text availability, unless no alternative source existed for key findings; (3) studies focused on pharmacological interventions unrelated to exercise physiology; and (4) non-peer-reviewed sources, including commercial websites and promotional materials.

### 2.4. Study Selection and Data Synthesis

The initial database search yielded 1105 records. After removal of duplicates, titles and abstracts were screened for relevance. Full-text articles were retrieved for all records considered potentially relevant. A total of 102 references were included in the final review.

Information was synthesized narratively and organized thematically into the following domains: (1) mitochondrial bioenergetics in equine skeletal muscle; (2) structural and functional features of equine muscle relevant to mitochondrial adaptation; (3) molecular mechanisms of exercise-induced mitochondrial remodeling; (4) metabolic plasticity and substrate utilization; (5) experimental evidence from equine studies; (6) practical implications for training; and (7) oxidative stress and maladaptive responses.

### 2.5. Classification of Evidence

Because this review integrates research from both equine and non-equine sources, the following classification framework was applied to ensure transparency regarding the origin and strength of evidence:

Direct equine evidence: original research conducted in horses, irrespective of breed or discipline.

Translational evidence: studies conducted in humans or rodent models with mechanistic relevance to equine skeletal muscle physiology.

General biological evidence: foundational biochemistry and cell biology applicable across mammalian species.

Throughout the text, the species origin of cited evidence is identified to allow the reader to distinguish between established equine findings and extrapolated knowledge. This distinction is particularly important given the limited number of mechanistic studies conducted directly in horses.

## 3. Mitochondria as the Bioenergetic Hub of Equine Skeletal Muscle

**Figure 1:**
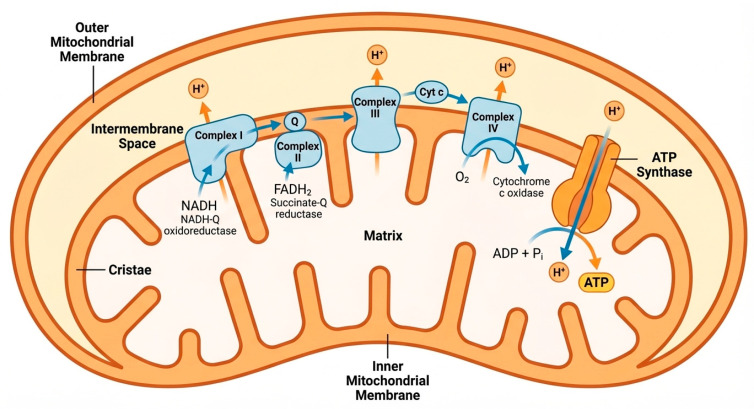
Structural organization of the mitochondrion and the oxidative phosphorylation system.

Mitochondria are multifunctional organelles responsible for integrating cellular metabolism with energy production [15]. Their primary role is the synthesis of ATP through oxidative phosphorylation, a process that occurs within the inner mitochondrial membrane [16]. This process involves the transfer of electrons through a series of protein complexes collectively known as the electron transport chain [17]. The structural organization of the mitochondrion and the main components of the oxidative phosphorylation system are illustrated in Figure 1.

Mitochondria generate ATP through oxidative phosphorylation within the inner mitochondrial membrane. Electrons derived from NADH and FADH_2_ are transferred through the electron transport chain complexes (I–IV), driving proton pumping into the intermembrane space. The resulting electrochemical gradient powers ATP synthesis through ATP synthase (Complex V). This figure was created by the authors using FigureLabs (Nano Banana Pro model, FigureLabs).

The electron transport chain consists of four major complexes (Complex I–IV) and ATP synthase (Complex V) [18]. Electrons derived from metabolic substrates are transferred through these complexes, driving the pumping of protons from the mitochondrial matrix into the intermembrane space. The resulting proton gradient generates an electrochemical potential that powers ATP synthesis [19,20].

Complex V (ATP synthase) represents the final step of oxidative phosphorylation and directly converts the energy stored within the proton motive force into ATP. The efficiency of ATP synthase is therefore a critical determinant of mitochondrial energy conversion efficiency. Alterations in ATP synthase activity have been associated with changes in aerobic performance and mitochondrial function across mammalian species [18,19,20].

In exercising skeletal muscle, ATP production is not limited by a single enzymatic step but rather by the integrated capacity of oxygen delivery, substrate availability, electron transport chain activity, and ATP synthase function. In both horses and other mammals, mitochondrial oxidative phosphorylation capacity and oxygen utilization become major determinants of sustained aerobic ATP production during prolonged exercise. Consequently, training-induced increases in mitochondrial density and respiratory capacity contribute directly to improvements in ATP turnover and aerobic performance [21,22,23].

During exercise, skeletal muscle ATP demand increases dramatically. Energy is required for several essential processes including the mechanical work of muscle contraction, active transport of calcium ions within muscle fibers, and the maintenance of ionic gradients across cellular membranes. These energy-consuming processes operate simultaneously during exercise, necessitating rapid and sustained ATP production [21,22,23].

Mitochondria integrate inputs from multiple metabolic pathways to meet these energy requirements. Carbohydrates are metabolized through glycolysis, producing pyruvate that enters mitochondria and is converted to acetyl-CoA by the pyruvate dehydrogenase complex [24,25]. Fatty acids undergo β-oxidation within the mitochondrial matrix, generating acetyl-CoA and reducing equivalents that feed into the electron transport chain [26].

The efficiency of these metabolic pathways determines the ability of skeletal muscle to sustain aerobic exercise. Increased mitochondrial density allows for greater oxidative capacity, enabling muscle fibers to produce ATP at higher rates while maintaining metabolic homeostasis [27,28].

In equine skeletal muscle, mitochondrial function is particularly important because locomotor muscles are capable of generating extremely high metabolic fluxes [14]. Horses performing maximal exercise exhibit rapid increases in oxygen consumption and metabolic turnover, placing considerable demands on mitochondrial oxidative capacity [14].

Beyond ATP production, mitochondria also regulate intracellular signaling processes. Reactive oxygen species produced during oxidative phosphorylation act as signaling molecules that modulate gene expression and activate adaptive pathways [29]. Although excessive ROS production can cause oxidative damage, moderate ROS levels contribute to beneficial adaptive responses that enhance mitochondrial function and antioxidant capacity [30].

Thus, mitochondria act not only as energy-producing organelles but also as metabolic sensors that coordinate cellular responses to exercise-induced stress. From a functional perspective, these mitochondrial properties are directly relevant to equine athletic performance because they determine the efficiency of ATP production, the ability to sustain oxidative metabolism during prolonged exercise, and the rate at which metabolic homeostasis can be restored after exertion [31].

## 4. Structural and Functional Features of Equine Skeletal Muscle Relevant to Mitochondrial Adaptation

**Figure 2:**
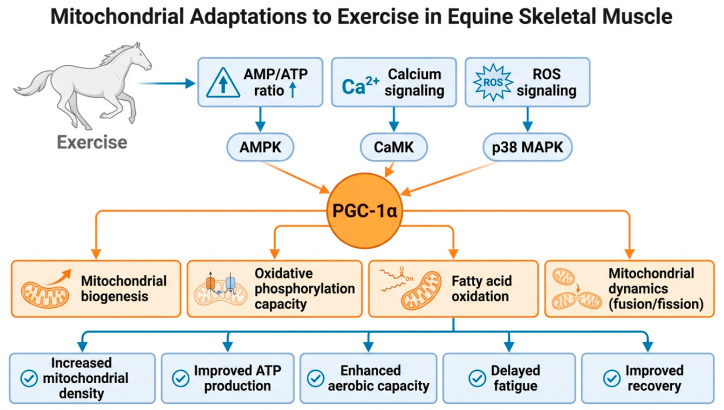
An overview of exercise-induced mitochondrial adaptation in equine skeletal muscle. Exercise activates metabolic and calcium-dependent signaling pathways including AMPK, CaMK, and p38 MAPK, which converge on the transcriptional coactivator PGC-1α. Activation of these pathways stimulates mitochondrial biogenesis, enhances oxidative phosphorylation capacity, and promotes metabolic remodeling. These adaptations ultimately increase mitochondrial density, improve ATP production efficiency, and contribute to enhanced aerobic performance and fatigue resistance in equine athletes. Created by the authors using FigureLabs.

The major pathways linking exercise stimuli to mitochondrial remodeling in equine skeletal muscle are summarized in Figure 2.

Equine skeletal muscle is highly specialized for athletic performance. Structural and metabolic characteristics of muscle fibers determine the ability of the muscle to sustain exercise and adapt to training stimuli [32].

Muscle fibers are commonly classified into three major types based on their contractile and metabolic properties: type I (slow-twitch oxidative), type IIA (fast oxidative–glycolytic), and type IIX (fast glycolytic). These fiber types differ substantially in mitochondrial content, oxidative enzyme activity, and fatigue resistance [33,34,35].

Type I fibers exhibit the highest mitochondrial density and oxidative capacity. These fibers contain abundant mitochondria and extensive capillary networks, enabling efficient oxygen delivery and sustained ATP production through oxidative metabolism. Type I fibers are particularly important for endurance exercise, where prolonged aerobic energy production is required [33].

Type IIA fibers display intermediate metabolic characteristics. They possess substantial mitochondrial content while maintaining the ability to generate rapid contractions. This combination of oxidative and glycolytic capacity makes type IIA fibers well suited for activities that require both power and endurance [33].

Type IIX fibers rely primarily on anaerobic glycolysis and have relatively low mitochondrial density. These fibers are capable of generating high levels of force but fatigue rapidly due to limited oxidative capacity [32,36,37].

The distribution of these fiber types varies between breeds and disciplines. Endurance horses typically possess higher proportions of oxidative fibers, whereas sprinting racehorses have greater proportions of glycolytic fibers [33,38,39].

Importantly, muscle fiber composition is not fixed. Exercise training can induce metabolic remodeling of skeletal muscle, including transitions between fiber types. One commonly observed adaptation is the shift from type IIX fibers toward type IIA fibers, which increases oxidative capacity and improves fatigue resistance [33,40,41].

In horses, these fiber type transitions are strongly influenced by training modality. Endurance-oriented conditioning generally promotes a shift toward a more oxidative phenotype, characterized by increased proportions of type IIA fibers and enhanced mitochondrial content. Conversely, sprint-oriented training tends to preserve fast-contracting characteristics while improving oxidative support within existing fiber populations. Mathematical and physiological models of muscle metabolism suggest that such adaptations improve substrate utilization efficiency, delay lactate accumulation, and enhance fatigue resistance by optimizing the balance between oxidative and glycolytic energy production [38,40,41,42,43].

In addition to fiber composition, mitochondrial distribution within muscle fibers plays an important role in metabolic performance [38,42]. Mitochondria are typically located in two major regions: subsarcolemmal mitochondria located beneath the cell membrane and intermyofibrillar mitochondria situated between contractile filaments. These populations may differ in functional characteristics and contribute differently to cellular metabolism [43,44].

### Mitochondrial Phenotype and Markers of Oxidative Capacity

The concept of the mitochondrial phenotype is particularly relevant in equine exercise physiology because it integrates both structural and functional aspects of mitochondrial metabolism. In practical terms, this phenotype includes mitochondrial density, oxidative enzyme activity, respiratory capacity, substrate utilization patterns, and the efficiency of mitochondrial quality control mechanisms [14,38,45].

In experimental studies, mitochondrial phenotype is commonly assessed using a combination of biochemical and functional markers. Citrate synthase activity is widely used as an indirect indicator of mitochondrial content, whereas enzymes such as succinate dehydrogenase and cytochrome c oxidase provide additional information about oxidative capacity [38,46]. More recently, high-resolution respirometry has enabled direct evaluation of mitochondrial respiratory function in equine skeletal muscle [14,47,48].

Distinguishing between mitochondrial content and intrinsic mitochondrial function is particularly important when interpreting exercise-induced adaptations. An increase in mitochondrial density does not necessarily imply that each individual mitochondrion becomes more efficient. Rather, training may initially increase the total oxidative potential of the muscle through expansion of mitochondrial mass, with functional refinement occurring later. This distinction has important implications for understanding both the timing and quality of adaptation in equine athletes [49,50].

In equine research, mitochondrial characteristics are frequently evaluated in the gluteus medius muscle, a major locomotor muscle involved in propulsion during galloping [47]. Because this muscle contributes significantly to force generation during exercise, mitochondrial adaptations in this muscle have direct implications for athletic performance. In horses, these markers are especially informative when evaluated in locomotor muscles that are directly involved in propulsion and performance, such as the gluteus medius [49,51].

## 5. Molecular Mechanisms of Exercise-Induced Mitochondrial Remodeling

Most mechanistic knowledge regarding AMPK, CaMK, p38 MAPK, PGC-1α signaling, mitochondrial dynamics, and mitophagy originates from studies in humans and rodent models. Direct equine evidence remains comparatively limited and is highlighted separately where available. These pathways integrate metabolic signals generated during exercise with transcriptional responses that modify mitochondrial structure and function [52,53].

Most signaling pathways described in this section are initially activated in response to a single exercise bout. However, the mitochondrial adaptations observed in trained horses result from the repeated activation of these signaling events over successive training sessions. Therefore, acute molecular signaling should be viewed as the initiating stimulus, whereas mitochondrial biogenesis and functional remodeling represent chronic adaptive outcomes of training [52,53,54,55,56].

One of the most important regulators of mitochondrial biogenesis is the transcriptional coactivator peroxisome proliferator-activated receptor gamma coactivator-1 alpha (PGC-1α). This protein acts as a master regulator of mitochondrial metabolism and coordinates the expression of genes involved in oxidative phosphorylation [54].

**Figure 3:**
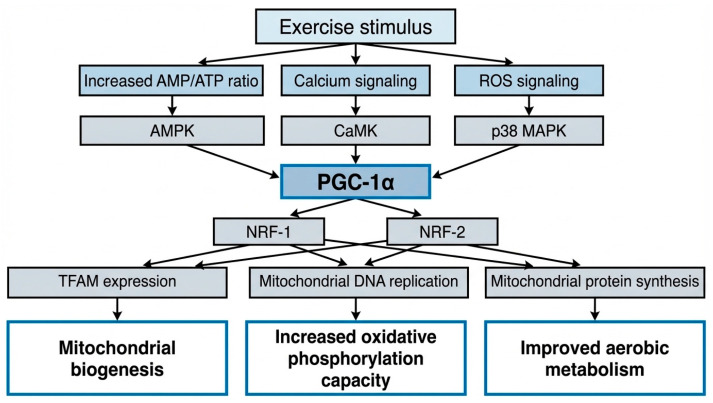
Molecular regulation of exercise-induced mitochondrial biogenesis in skeletal muscle. Exercise activates metabolic and calcium-dependent signaling pathways including AMP-activated protein kinase (AMPK), calcium/calmodulin-dependent kinase (CaMK), and p38 MAPK. These pathways converge on the transcriptional coactivator PGC-1α, which regulates nuclear respiratory factors (NRF-1 and NRF-2) and mitochondrial transcription factor A (TFAM). Activation of this signaling cascade stimulates mitochondrial DNA replication and the expression of mitochondrial proteins, ultimately promoting mitochondrial biogenesis and enhanced oxidative metabolism. Created by the authors using FigureLabs.

Exercise increases the AMP/ATP ratio within muscle cells, activating AMP-activated protein kinase (AMPK) [55]. Activation of AMPK signals cellular energy stress and promotes metabolic pathways that increase ATP production [56]. At the same time, muscle contraction elevates intracellular calcium levels, activating calcium-dependent kinases such as CaMK [57]. The molecular signaling pathways regulating exercise-induced mitochondrial biogenesis in skeletal muscle are summarized in Figure 3.

These signaling pathways converge on PGC-1α, which activates transcription factors including nuclear respiratory factors (NRF-1 and NRF-2). These transcription factors regulate genes encoding components of the electron transport chain and other mitochondrial proteins [58].

PGC-1α also promotes the expression of mitochondrial transcription factor A (TFAM), which is essential for the replication and transcription of mitochondrial DNA. Through these coordinated mechanisms, exercise stimulates the synthesis of new mitochondria and expands the mitochondrial network [59].

In addition to mitochondrial biogenesis, exercise induces mitochondrial dynamics, which involve the fusion and fission of mitochondrial networks. Fusion allows mitochondria to exchange metabolic substrates and mitochondrial DNA, helping maintain functional integrity [60]. Fission facilitates redistribution of mitochondria within muscle fibers and enables removal of damaged mitochondrial segments [61].

Another important mechanism of mitochondrial quality control is mitophagy, the selective degradation of dysfunctional mitochondria through autophagic pathways. Mitophagy prevents accumulation of damaged mitochondria that could impair cellular metabolism [62].

Reactive oxygen species (ROS) also play an important role in mitochondrial adaptation to exercise. Although excessive ROS production can lead to oxidative damage, moderate ROS generation acts as a signaling mechanism that stimulates mitochondrial biogenesis and antioxidant defense systems [63,64]. Exercise-induced ROS signaling has been shown to activate pathways involved in mitochondrial remodeling and metabolic adaptation in skeletal muscle [65].

Together, mitochondrial biogenesis, dynamics, and mitophagy contribute to continuous remodeling of the mitochondrial network in response to exercise. Collectively, these molecular mechanisms increase the capacity of skeletal muscle to sustain oxidative ATP production, improve resistance to metabolic stress, and support the functional adaptations required for athletic performance in horses [52].

## 6. Metabolic Plasticity and Substrate Utilization During Exercise

**Figure 4:**
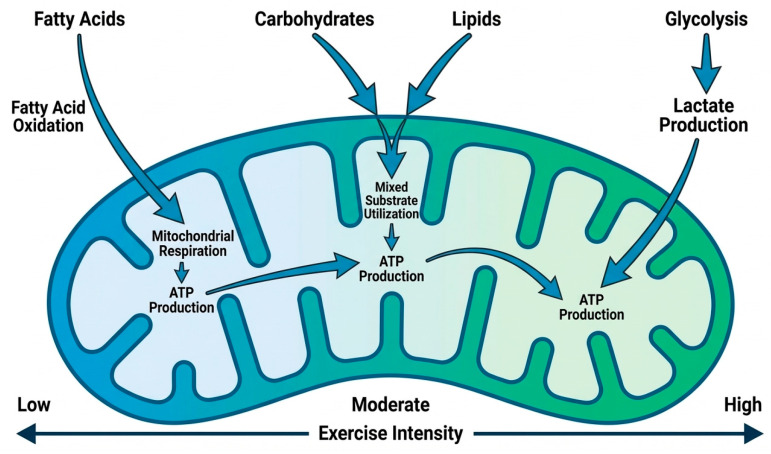
Metabolic substrate utilization during exercise in equine skeletal muscle. During low-intensity exercise, fatty acid oxidation represents the dominant source of ATP production through mitochondrial oxidative metabolism. As exercise intensity increases, carbohydrate metabolism becomes progressively more important due to the faster rate of ATP generation through glycolysis. Training enhances metabolic flexibility, enabling efficient switching between lipid and carbohydrate substrates. Created by the authors using FigureLabs.

One of the most important characteristics of skeletal muscle adaptation to exercise is its capacity for metabolic plasticity, defined as the ability to adjust substrate utilization in response to changes in energy demand [66]. During exercise, skeletal muscle must continuously balance the oxidation of carbohydrates, fatty acids, and, to a lesser extent, amino acids in order to sustain ATP production [67,68]. The relative contribution of these substrates depends on exercise intensity, duration, training status, and nutritional availability. The dynamic shift in substrate utilization during exercise is illustrated in Figure 4.

At lower exercise intensities, fatty acids represent the primary substrate for mitochondrial oxidation [69]. Fatty acid oxidation produces large quantities of ATP and is therefore well suited for sustained aerobic activity [70]. In horses performing prolonged exercise, enhanced fatty acid oxidation allows for efficient ATP generation while preserving limited glycogen stores. This mechanism is particularly important in endurance disciplines, where maintaining energy availability over extended periods is essential for performance [71].

As exercise intensity increases, the relative contribution of carbohydrate metabolism becomes greater [72]. Glycolysis provides ATP more rapidly than fatty acid oxidation, allowing skeletal muscle to sustain higher power output. However, increased reliance on glycolysis leads to the accumulation of metabolic byproducts such as lactate and hydrogen ions, which may contribute to fatigue if oxidative metabolism cannot keep pace with energy demand [73].

Training induces significant modifications in substrate utilization patterns. Endurance training increases the expression of enzymes involved in fatty acid transport and β-oxidation, enhancing the capacity of skeletal muscle to utilize lipid substrates. In addition, mitochondrial density and respiratory capacity increase, allowing for greater oxidative metabolism of both carbohydrates and lipids [74,75].

Improved mitochondrial function also enhances the oxidation of lactate within skeletal muscle. Lactate, once considered merely a metabolic waste product, is now recognized as an important metabolic intermediate that can be oxidized within mitochondria. Increased mitochondrial density allows for more efficient lactate utilization, contributing to improved metabolic efficiency during exercise [76].

Metabolic plasticity also plays a key role in determining the lactate threshold, a physiological parameter widely used to assess athletic performance. The lactate threshold represents the exercise intensity at which lactate production exceeds its rate of clearance. Horses with higher mitochondrial oxidative capacity can sustain higher exercise intensities before reaching this threshold, allowing for superior aerobic performance [77,78].

Furthermore, mitochondrial adaptations influence metabolic recovery following exercise. Enhanced oxidative capacity accelerates the restoration of ATP and phosphocreatine levels, facilitates lactate clearance, and promotes the re-synthesis of glycogen stores [79,80]. These processes are essential for maintaining performance during repeated exercise bouts.

In equine athletes, metabolic plasticity therefore represents a key determinant of endurance capacity, fatigue resistance, and recovery efficiency. Mitochondrial remodeling induced by training plays a central role in supporting this metabolic flexibility [81].

Nutritional factors may further modulate mitochondrial adaptation in equine skeletal muscle. Dietary antioxidants, mitochondrial cofactors, and nutrient availability may influence oxidative metabolism, redox balance, and post-exercise recovery [82]. Although evidence in horses remains limited, these interventions may represent an additional layer through which mitochondrial phenotype can be shaped in athletic animals [49].

## 7. Experimental Evidence in Horses

Although the majority of mechanistic insights into mitochondrial biology originate from studies conducted in humans and laboratory animals, several experimental studies have investigated mitochondrial adaptations in equine skeletal muscle. A summary of key experimental studies investigating mitochondrial adaptations to exercise in horses is presented in Table 1.

Importantly, most equine studies summarized in this review evaluate mitochondrial characteristics following weeks or months of structured conditioning programs. Consequently, the reported increases in mitochondrial content, respiratory capacity, and oxidative function should be interpreted as chronic training adaptations rather than acute responses to individual exercise sessions.

One of the earliest approaches used to investigate mitochondrial function in horses involved the measurement of oxidative enzyme activity in muscle biopsies [14]. Enzymes such as citrate synthase and succinate dehydrogenase serve as markers of mitochondrial density and oxidative capacity. Studies conducted in Thoroughbred racehorses have consistently demonstrated increases in these enzymatic activities following structured training programs, indicating enhanced mitochondrial content [76,83,84].

More recent investigations have utilized high-resolution respirometry to directly measure mitochondrial respiratory function in muscle tissue samples. These techniques allow for detailed analysis of mitochondrial oxidative phosphorylation and provide insight into the functional capacity of the electron transport chain. Research using this approach has demonstrated that training improves mitochondrial respiratory capacity in locomotor muscles of Thoroughbred horses [14,85].

Interestingly, some studies have observed that although mitochondrial density increases during training, the intrinsic efficiency of individual mitochondria may not increase proportionally. This observation suggests that early adaptations to training may involve rapid expansion of mitochondrial mass rather than immediate improvements in mitochondrial efficiency [11,86].

Longitudinal studies examining mitochondrial development in young Thoroughbred horses have shown that mitochondrial volume density increases during the early stages of training [49]. However, intrinsic mitochondrial function measured per unit of mitochondrial mass may decrease slightly during this period. This phenomenon may reflect structural remodeling of the mitochondrial network as the muscle adapts to increased metabolic demands [9,11].

Other studies comparing skeletal and cardiac muscle have reported that skeletal muscle exhibits greater adaptive changes during training. Cardiac muscle already possesses high mitochondrial density and oxidative capacity under resting conditions, limiting the magnitude of further adaptation [81].

Experimental research has also investigated the effects of nutritional interventions on mitochondrial function in horses. For example, supplementation with mitochondrial cofactors such as coenzyme Q10 has been associated with changes in skeletal muscle respiration and antioxidant capacity. These findings suggest that nutritional strategies may influence mitochondrial adaptation during training [87].

Despite these advances, experimental studies on mitochondrial physiology in horses remain relatively limited compared with research conducted in humans. Nevertheless, available evidence clearly indicates that mitochondrial remodeling plays a central role in the physiological adaptations that underlie improved athletic performance in equine athletes. These findings highlight the importance of mitochondrial function as a determinant of aerobic capacity and training adaptation in horses. Understanding how mitochondrial remodeling occurs during different phases of training may help optimize conditioning programs and improve performance outcomes in equine athletes. Overall, the available equine literature consistently supports an increase in mitochondrial content and oxidative capacity in response to training. However, the evidence remains limited by small sample sizes, heterogeneity in training protocols, differences in sampled muscles, and methodological variability in mitochondrial assessment. These limitations complicate direct comparisons among studies and may partly explain the discrepancies observed between markers of mitochondrial content and intrinsic mitochondrial function.

### Practical Implications for Training and Performance

From a practical perspective, mitochondrial adaptations are highly relevant for conditioning strategies in equine athletes. Training programs that progressively increase aerobic workload are more likely to stimulate mitochondrial biogenesis, improve oxidative phosphorylation capacity, and enhance metabolic flexibility without exceeding the adaptive capacity of the locomotor apparatus [11,49].

Improved mitochondrial function is expected to translate into better oxygen utilization, delayed lactate accumulation, greater fatigue resistance, and more efficient post-exercise recovery. These effects are particularly relevant in disciplines that require sustained aerobic effort, repeated high-intensity bouts, or rapid recovery between training sessions [48,88,89].

At the same time, the benefits of mitochondrial adaptation must be interpreted alongside the slower structural adaptation of tendons, ligaments, and bone [10,90]. A rapid increase in metabolic capacity may allow horses to perform at higher intensities before connective tissues have fully adapted to the corresponding mechanical loads [91]. Consequently, training design should account not only for cardiovascular and metabolic readiness, but also for the biomechanical resilience of the locomotor system [92].

From a practical standpoint, trainers may use progressive aerobic conditioning to stimulate mitochondrial biogenesis while minimizing excessive musculoskeletal loading. Monitoring variables such as heart rate recovery, lactate threshold, and recovery kinetics may provide indirect indicators of mitochondrial adaptation. Furthermore, gradual workload progression is particularly important in young horses, where mitochondrial adaptations may develop faster than tendon, ligament, and bone remodeling [11,48,49,88,92].

Taken together, current evidence suggests that mitochondrial physiology may provide a useful framework for optimizing conditioning, recovery scheduling, and workload progression in sport horses.

## 8. From Adaptation to Maladaptation: Oxidative Stress, Recovery, and Overuse Risk

**Figure 5:**
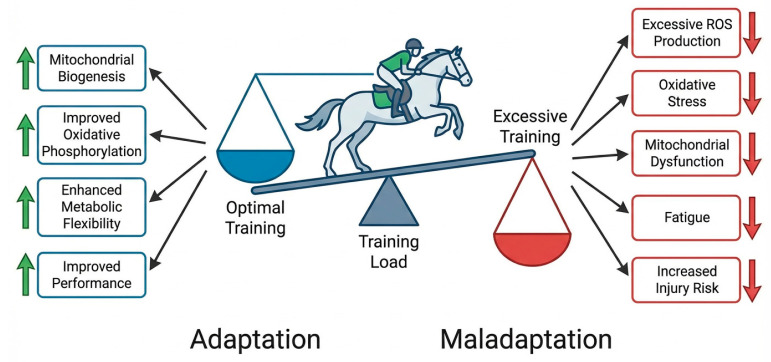
The balance between beneficial mitochondrial adaptation and maladaptive responses to training. Moderate training stimulates mitochondrial biogenesis, improves oxidative metabolism, and enhances aerobic performance. In contrast, excessive training loads combined with insufficient recovery may increase reactive oxygen species production and oxidative stress, potentially leading to mitochondrial dysfunction, fatigue, and increased risk of musculoskeletal injury. Created by the authors using FigureLabs.

While mitochondrial adaptations are generally beneficial for performance, excessive exercise or insufficient recovery may lead to maladaptive responses. One of the primary mechanisms underlying these effects is oxidative stress [93]. The balance between beneficial mitochondrial adaptation and potential maladaptive responses associated with excessive training load is summarized in Figure 5.

During oxidative phosphorylation, small amounts of reactive oxygen species are inevitably generated as byproducts of electron transport. Under physiological conditions, antioxidant systems within the cell neutralize these molecules and maintain redox balance. However, intense or prolonged exercise can increase mitochondrial ROS production beyond the capacity of antioxidant defenses [94,95].

Excessive ROS accumulation may damage mitochondrial proteins, lipids, and DNA, impairing mitochondrial function and reducing ATP production [96]. Oxidative stress may also activate inflammatory pathways that contribute to muscle fatigue and tissue damage [97].

Recovery periods between exercise sessions are therefore essential for restoring cellular homeostasis and allowing adaptive processes to occur. Adequate recovery promotes mitochondrial repair, removal of damaged organelles through mitophagy, and synthesis of new mitochondrial proteins [10,49,98].

Another important factor in the transition from adaptation to maladaptation is the relationship between metabolic and structural adaptation. Skeletal muscle can adapt relatively rapidly to increased workloads through. In contrast, connective tissues such as tendons and ligaments adapt more slowly because of lower vascularization and slower collagen turnover [81,99].

As a result, improvements in muscular metabolic capacity may outpace the structural adaptation of the locomotor system. This mismatch can increase the risk of musculoskeletal injuries, particularly in horses undergoing intensive training programs [10].

In equine athletes, common overuse injuries include tendonitis, suspensory ligament damage, and stress-related bone injuries. These conditions often arise when training intensity increases too rapidly or when recovery periods are insufficient [100,101].

Understanding the relationship between mitochondrial adaptation, oxidative stress, and tissue remodeling is therefore essential for developing training programs that optimize performance while minimizing injury risk.

## 9. Knowledge Gaps and Future Directions

Despite growing interest in mitochondrial biology in equine exercise physiology, several important gaps remain in the current literature.

First, many molecular mechanisms regulating mitochondrial adaptation have been characterized primarily in human or rodent models. Although these findings provide valuable insights, species-specific differences may exist in horses. Further research is needed to determine whether regulatory pathways identified in other species operate similarly in equine skeletal muscle [5,11].

Second, many studies investigating mitochondrial function in horses rely on relatively small sample sizes due to logistical challenges associated with large animal research. Expanding the scale of experimental studies would improve statistical power and allow for better evaluation of variability among breeds, training programs, and athletic disciplines [102].

Third, most available research focuses on relatively short training periods. Long-term longitudinal studies examining mitochondrial adaptations over entire training seasons or across different stages of a horse’s athletic career would provide valuable insights into the dynamics of mitochondrial remodeling.

### Methodological Limitations of Current Equine Studies

A major limitation of the current equine literature is the heterogeneity of experimental design. Studies differ substantially in breed, age, training history, exercise modality, muscle sampled, and analytical methods used to assess mitochondrial adaptation. This variability complicates direct comparison across studies and may partly explain the inconsistencies observed between markers of mitochondrial content and intrinsic mitochondrial function.

Another important limitation is that many studies rely on indirect biochemical markers of mitochondrial adaptation, whereas fewer investigations incorporate direct functional techniques such as high-resolution respirometry. In addition, longitudinal data remain scarce, making it difficult to determine the temporal sequence of mitochondrial remodeling during different phases of training.

An additional limitation is the scarcity of mechanistic studies performed directly in horses. Ethical considerations, the invasive nature of repeated muscle biopsies, high maintenance costs, and limited availability of specialized research facilities restrict the feasibility of large-scale mechanistic investigations. Consequently, many molecular pathways discussed in the present review are inferred from human and rodent studies. While these translational models provide valuable biological insights, species-specific differences in muscle fiber composition, exercise physiology, and metabolic regulation warrant caution when extrapolating findings to equine athletes.

Standardization of biopsy protocols, respiratory measurements, and performance-related endpoints would greatly improve the comparability of future studies and strengthen the evidence base for practical application in equine sports medicine.

Emerging technologies offer promising opportunities for addressing these questions. Advances in omics approaches, including transcriptomics, proteomics, metabolomics, and epigenomics, allow for comprehensive analysis of molecular responses to exercise. Integrating these approaches with physiological measurements such as high-resolution respirometry and performance testing could significantly enhance our understanding of mitochondrial adaptations in equine athletes.

Finally, research integrating molecular biology with practical training strategies could help translate scientific findings into improved performance outcomes. Understanding how training intensity, duration, and recovery influence mitochondrial adaptation may lead to more effective and individualized training programs for equine athletes.

Addressing these research gaps will be essential for developing a comprehensive understanding of mitochondrial physiology in equine athletes and for translating molecular insights into practical applications in equine sports medicine.

Future studies should also investigate the role of mitochondrial dysfunction in equine myopathies and other muscle disorders, as alterations in mitochondrial quality control and oxidative metabolism may contribute to disease pathogenesis and impaired athletic performance.

## 10. Conclusions

Mitochondrial adaptations are a key part of the physiological response to exercise in horses. Processes such as mitochondrial biogenesis, respiratory remodeling, metabolic flexibility, and quality control enable skeletal muscle to meet increased energy demands during training. These changes enhance oxidative metabolism, delay fatigue, and support efficient recovery, making mitochondrial remodeling central to aerobic performance.

Future research integrating molecular biology, exercise physiology, and sports medicine will further clarify the role of mitochondrial function in equine athletic performance. Such knowledge may ultimately contribute to the development of more effective training strategies and improved welfare for equine athletes.

## Figures and Tables

**Table 1:** Summary of experimental studies investigating mitochondrial adaptations to exercise in horses.

Study	Horse Population	Methodology	Main Findings
Votion et al. (2012) [14]	Healthy horses	High-resolution respirometry	Mitochondrial respiratory capacity correlates with aerobic fitness
Wesolowski et al. (2021) [13]	Healthy horses subjected to training protocols	Muscle biopsy and enzymatic assays	Training increases mitochondrial density and oxidative capacity
White et al. (2017) [11]	American Quarter Horse yearlings	9-week submaximal exercise training, muscle biopsies, high-resolution respirometry (HRR), and enzymatic assays	Submaximal training improved mitochondrial function/efficiency in the gluteus medius, but did not increase mitochondrial content
Latham et al. (2021) [9]	Young vs. aged horses	Muscle biopsies and mitochondrial function analysis	Exercise improves mitochondrial capacity, but aged horses show impaired mitochondrial function and altered adaptation

## Data Availability

No new data were created or analyzed in this study. Data sharing is not applicable to this article.

## Abbreviations

The following abbreviations are used in this manuscript:

AMPK	AMP-activated protein kinase
ATP	adenosine triphosphate
CaMK	calcium/calmodulin-dependent kinase
NRF	nuclear respiratory factor
OXPHOS	oxidative phosphorylation
PGC-1α	peroxisome proliferator-activated receptor gamma coactivator-1 alpha
ROS	reactive oxygen species
TFAM	mitochondrial transcription factor A
